# The Importance of an Early Evaluation after Establishing a Gluten-Free Diet in Children with Celiac Disease

**DOI:** 10.3390/nu15071761

**Published:** 2023-04-04

**Authors:** Rafael Martín-Masot, María Jiménez-Muñoz, Marta Herrador-López, Marta Flor-Alemany, Víctor Manuel Navas-López, Teresa Nestares

**Affiliations:** 1 Pediatric Gastroenterology and Nutrition Unit, Hospital Regional Universitario de Malaga, 29010 Málaga, Spain; 2 Institute of Nutrition and Food Technology “José MataixVerdú” (INYTA), Biomedical Research Centre (CIBM), University of Granada, 18071 Granada, Spain; 3 Department of Physiology, Faculty of Pharmacy, University of Granada, 18071 Granada, Spain

**Keywords:** celiac disease, gluten-free diet, gluten immunogenic peptides, ultra-processed foods

## Abstract

A gluten-free diet (GFD) is the only treatment available for celiac disease (CD); hence, it is important to ensure correct adherence to the diet and adequate monitoring of the diet. The present study aims to assess the importance of an early follow-up of celiac patients after diagnosis of the disease, identify the role of stool gluten immunogenic peptides (GIPs) in the assessment of GFD adherence, and analyze possible nutritional imbalances or deficiencies in the GFD. This is a cross-sectional study carried out in pediatric patients with newly diagnosed CD in a tertiary hospital in Spain. Of the 61 patients included, 14% had positive stool GIPS at 4 months after CD diagnosis, Among them, 88% had negative stool GIPS at 9 months after diagnosis, following dietary advice. We found nutritional deficiencies in the GFD, such as vitamin D (with only 27% of patients with adequate intakes), folate, calcium, magnesium, and fiber. Similarly, we found imbalances: excess protein and fat intakes and a high percentage of total daily energy intake came from ultra-processed foods (UPF). These findings emphasize the importance of early follow-up of children after diagnosis of CD. It is also crucial to identify patients with poor GFD compliance based on stool GIPS and analyze GFD nutritional imbalances and deficits. Our findings may contribute to the development of specific strategies for the early follow-up of patients with CD, including appropriate nutritional counselling.

## 1. Introduction

Celiac disease (CD) is an immune-mediated systemic pathology, which appears and develops from the dysfunctional interaction between genetic and environmental factors [1]. It is characterized by damage of varying intensity in the duodenal mucosa and a heterogeneous combination of gastrointestinal and/or extraintestinal symptoms, with a prevalence of approximately 1.4% of the population [2,3,4] and an increasing incidence worldwide [5].

A strict lifelong gluten-free diet (GFD) is the only treatment currently available for CD patients. However, adherence to the GFD is difficult due to the large number of gluten-containing products and cross-contamination [6]. Socioeconomic aspects also play an important role. These factors lead to minimal adherence rates of up to 23% of children [7] and 53% of adults with CD [8]. Some studies in adults [9] have shown a high percentage (approximately 20%) of patients with non-responsive CD, and this is largely due to poor adherence to the GFD. Non-compliance with the GFD may contribute, in addition to enteropathy and, in some cases, persistent symptoms, to the development of diseases with high morbidity and high health and social costs, such as autoimmune diseases, osteopenia/osteoporosis, infertility, repeated miscarriages, neurological/psychiatric disorders, and cancer (especially of the gastrointestinal tract) [2,3,10].

Proper GFD monitoring is crucial, especially in the first year after diagnosis of the CD when adherence to GFD is essential for mucosal recovery. However, there are no firm recommendations on the most efficient method to assess adherence to GFD [11]. Once the GFD is established, traditional methods’ sensitivity (clinical, dietary records, and serological tests) decreases markedly to detect persistent mucosal damage [12] and may be ineffective in revealing dietary transgressions or the ingestion of trace of gluten [3]. Duodenal biopsies are reserved for refractory cases due to their invasiveness and cost.

This fact has led to the exploration of more sensitive methods for detecting dietary transgressions, and the multiple determination of gluten immunogenic peptides (GIPs) in biological samples (urine and/or stool) has emerged in this context [13]. GIPs are resistant to gastrointestinal digestion, and part of them can be absorbed into the bloodstream and subsequently excreted in the urine, so that their presence can be determined in urine and stool [14,15] within 6–12 h and 3–5 days, respectively, after gluten ingestion in a normal diet [16]. This non-invasive method provides direct information on recent gluten exposure [10]. Previous studies have evaluated its importance not only as a suitable method for detecting refractory CD [17] but also as a tool for monitoring adherence to GFD [18]. The presence of repeated positive GIPS over several days has been correlated with intestinal mucosal damage [19]. Moreover, its assessment in conjunction with a dietary record concomitant with sample collection allows targeted dietary interventions to be developed during patient follow-up [15,20,21].

The nutritional adequacy of GFD is a controversial issue, as the exclusion of gluten-containing cereals from the diet may lead to deficiencies in the B vitamin group, folic acid, fiber, and vitamin D [22]. Other notable deficiencies include magnesium, calcium, iron, and zinc deficiencies [22]. Therefore, nutritional deficiencies may be due not only to malabsorption caused by the disease itself but also to the characteristics of an inadequate GFD [23]. Recent studies [24] have shown that celiac patients have higher intakes of total fat and added sugars; however, it is unclear whether they have higher intakes of total energy. Most of the studies carried out so far [22,24,25] analyze GFD nutritional imbalances and deficits in the medium and long-term follow-up of patients with CD, and there is still a certain lack of knowledge about the nutritional adequacy and the best follow-up strategy of the GFD in newly diagnosed patients with CD. The nutritional imbalances that have been described in several studies [22,23,24] indicate that an early approach to the GFD follow-up could be essential, and it is necessary to establish the ideal time to initiate follow-up after diagnosis of the CD.

Consequently, the aims of the present study were to evaluate the importance of an early CD follow-up: (1) determine the adherence to the GFD in recently diagnosed patients with CD and (2) analyze possible nutritional imbalances or deficiencies of the GFD.

## 2. Materials and Methods

### 2.1. Subjects

A total of 61 children aged between 2 and 14 years with CD were included in the present cross-sectional pilot study. Participants were recruited from January 2021 to July 2022; children were diagnosed with CD according to the criteria of the European Society of Pediatric Gastroenterology Hepatology and Nutrition (ESPGHAN) [11] and received care at the Pediatric Gastroenterology and Nutrition Unit of the Hospital Regional Universitario in Malaga, Spain. Exclusion criteria were liver or kidney disease, acute and chronic inflammation, inflammatory bowel disease, diabetes, chronic asthma, and intake of dietary supplements containing substances with antioxidant activity.

The study was approved by the Ethics Committee of the Hospital Regional Universitario de Malaga (Ref. 0255-N-22). It was performed in accordance with the Declaration of Helsinki principles and its subsequent amendments. The clinical and sociodemographic characteristics of the participants were assessed by the same group of investigators. Informed consent was obtained from all subjects participating in the study.

### 2.2. Stool Collection and Analysis

After four months following a GFD, participants were instructed to collect two stool samples on non-consecutive days, one on weekdays and the other during the weekend. Participants were provided with stool collection materials, including special plastic containers with crew caps labels, cold bags, isothermal boxes, and cold packs, and were instructed to collect at least 10 g of stool each time and to record the date and time of collection. All stool samples were stored in isothermal boxes with cold packs at 4–8 °C and sent to the laboratory within 48 h of collection. All samples were stored at −20 °C until they were processed. If any of these samples were positive, participants were asked to collect two new samples after 9 months of the CD diagnosis.

The concentration of GIPs in stool samples was measured using a lateral flow technique with the iVYCHECK GIP Stool kit (Biomedal S.L., Seville, Spain) based on the manufacturer’s guidelines. This is a rapid immunochromatography test that detects GIPs in stool samples, with the possibility of a positive or negative result. This technique has demonstrated a sensitivity range of 95–100% and a specificity of 100% in several studies [26,27].

### 2.3. Anthropometric Measures

A scale and stadiometer (Seca 22, Hamburg, Germany) were employed to measure weight (kg) and height (m), respectively. Body mass index was calculated as (weight [Kg]/height [m^2^]).

### 2.4. Dietary Assessment

A three-day record was employed to assess dietary intake. Participants received instructions from a trained dietitian to guarantee proper handling of the dietary. In addition, they received a photographic atlas with a chart of household measures and a list of portion sizes [28]. All of the meals consumed throughout the day were included in the survey, along with a detailed description of the food’s quantity consumed (using the photographic atlas as a guide), its preparation (including cooking methods and sugar or fats added), and the brands of packaged foods consumed.

The Evalfinut 2.0 software, which includes the Spanish Food Composition Database [29], was used to analyze all diaries. The estimated energy (kilocalories) and macronutrient intake (measured in grams and including proteins, total fats, saturated fats, carbohydrates, simple sugar, and fiber), as well as the proportion of energy provided by each macronutrient, was calculated. Reference values for energy and nutrient consumption were drawn from the recommended energy mentioned previously and nutrient intake levels for the Spanish population [28]. The nutritional information on the labels of gluten-free products allowed us to determine the composition of these products.

Food was categorized using the NOVA categorization into four groups: unprocessed or minimally processed foods; processed culinary ingredients; processed foods; and ultra-processed foods [30]. It is the most widely used method for examining diets according to food processing and has been widely used by international agencies such as PAHO, WHO, and FAO [31,32,33].

In the event of a positive result in any of the samples delivered after 4 months of GFD, the team’s dietitian made a phone call visit, which included a nutritional education session. During the visit, a review of the detailed revision of food and gluten-free products described in the three-day record was carried out, and the concern about any possible doubts about GFD and cross-contamination was addressed.

### 2.5. Statistical Analyses

The baseline characteristics of the study sample were described using descriptive statistics (mean standard deviation) for quantitative variables and the percentage of participants (%) for categorical variables. The Chi-square test was additionally used to explore differences in categorical variables.

A one-way analysis of covariance (ANOVA) after adjustment for age, sex, and BMI was employed to assess differences in food group consumption and NOVA food classification of children by fecal GIP detection (negative vs. positive). After controlling for age, sex, and BMI, ANOVA was used to assess dietary intakes by the percentage of energy consumed from UPF in children with CD (below 50% of daily energy intake vs. above 50% of daily calorie intake). The dietary intakes were compared by ANOVA after adjusting for age, sex, and BMI.

The Statistical Package for Social Sciences (IBM SPSS Statistics for Windows, Version 22. IBM Corp, Armonk, NY, USA) was used to analyze the data, and the statistical significance was set at *p* < 0.05.

## 3. Results

Characteristics of the study sample are displayed in Table 1. A total of 61 children with CD participated in the study (mean age 7.5 ± 3.9 years). More than half of the participants were norm weight (72%) and did not have other diseases (94%).

**Figure 1:**
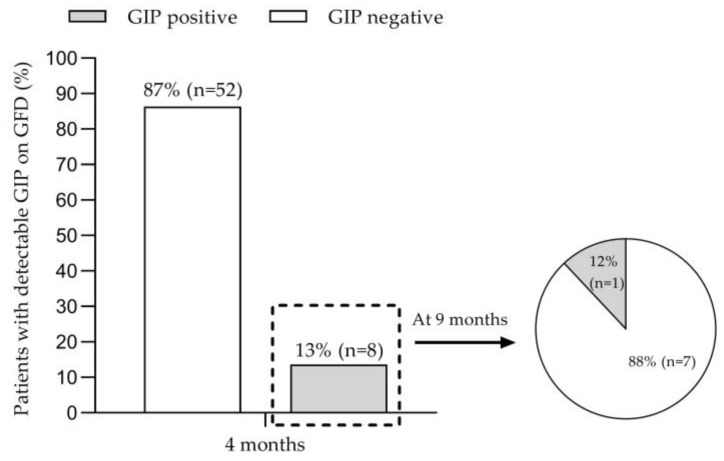
Stool gluten immunogenic peptide detection according to months following a gluten-free diet. GFD, gluten-free diet.

**Figure 2:**
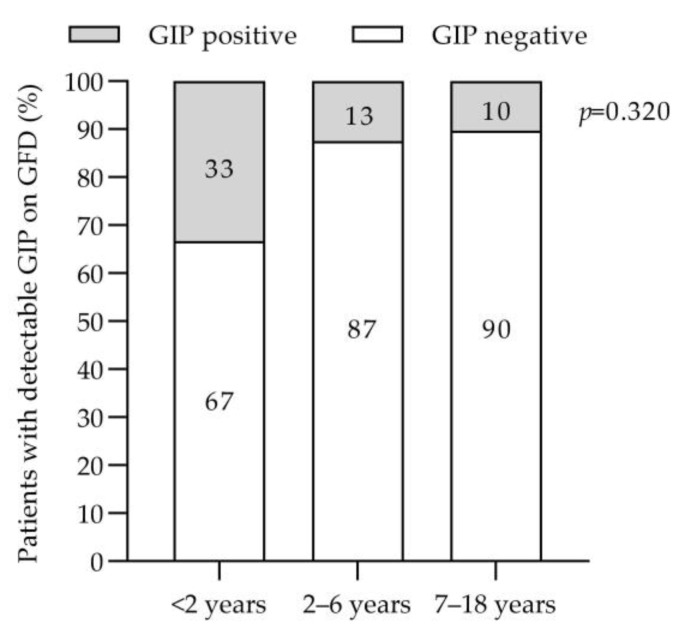
Stool gluten immunogenic peptide detection according to patient age. GIP, gluten immunogenic peptides. GFD, gluten-free diet.

The percentage of participants with stool GIP detection according to months on a GFD are shown in Figure 1. After 4 months following a GFD, eight patients (13.3%) tested positive for stool GIP (GIP+), and 52 patients (86.7%) tested negative (GIP−). After 9 months following a GFD, one child remained GIP+. Specifically, GIPs were detected in the stools of 33% of children with CD before 2 years of age and of 13% and 10% of children diagnosed at 2–6 years and at an older age, 7–18 years, respectively (Figure 2).

**Figure 3:**
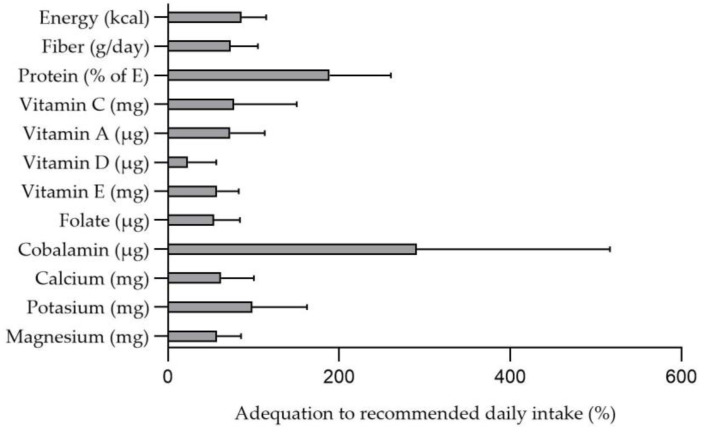
Percentage of adequacy for energy intake, fiber, protein, and micronutrients, recommended daily intake in children with celiac disease (n = 58).

**Figure 4:**
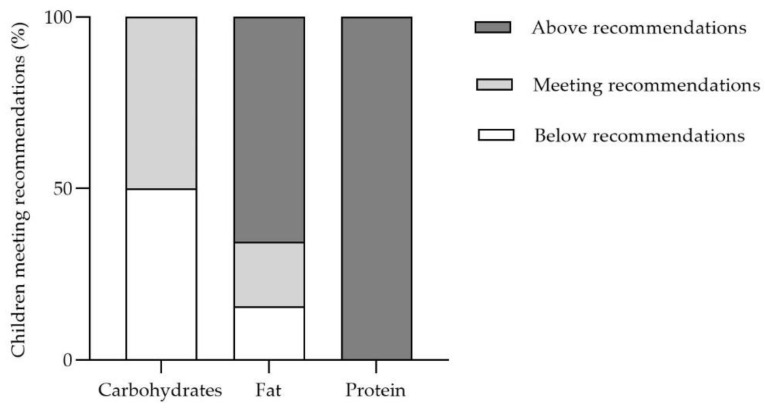
Children meeting European Food Safe Authority recommendations for carbohydrates, fat, and protein daily intakes.

The percentage of adequacy for energy, protein, and micronutrient profile intake by age group and sex according to Moreiras et al. [29] and other groups’ recommendations [34] is shown in Figure 3. The adequacy for energy, fiber, and protein intake was 89%, 73%, and 189%, respectively. Regarding micronutrients, the adequacy for Vitamin D was 23%, the adequacy for folate was 53%, and the adequacy for calcium and magnesium was 62% and 57%, respectively. Furthermore, according to the recommendations by the European Food Safety Authority (EFSA) [35], the percentage of children that meet the carbohydrate and protein intake was 50% and 19%, respectively (Figure 4). A total of 66% of children exceed the recommended intake for fat. Additionally, all children (100%) exceeded the recommended intake for protein. No differences in dietary intake and NOVA food classification by fecal GIP detection (GIP− vs. GIP+) were found (Table 2) (all *p* > 0.05). There were no significant differences in the percentage of adequacy according to fecal GIP detection (GIP+ versus GIP−) (*p* > 0.05) (Supplementary Figure S1).

**Figure 5:**
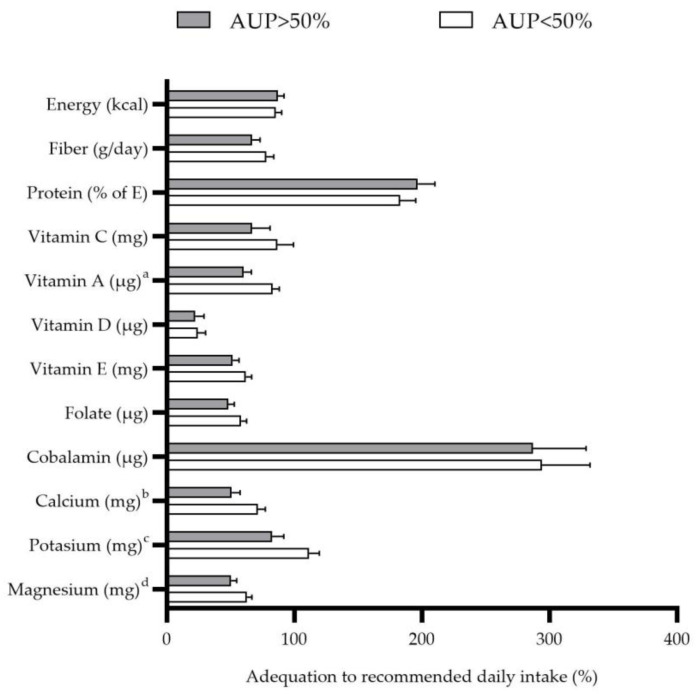
Percentage of adequacy for energy intake, fiber, protein, and micronutrients, recommended daily intake in children with celiac disease according to percentage of energy intake from ultra-processed food (n = 58). Values are shown as mean (standard error). Model is adjusted for age, sex, and body mass index (BMI). ^a^ *p* = 0.009; ^b^ *p* = 0.027; ^c^ *p* = 0.023; ^d^ *p* = 0.046.

The percentage of adequacy for energy intake, fiber, protein, and micronutrients, recommended daily intake by a percentage of daily energy consumed from UPF in celiac children, is shown in Figure 5. The group with the highest intake of energy from UPF (above 50% of total energy) showed a lower intake of vitamin A (*p* = 0.009), calcium (*p* = 0.027), potassium (*p* = 0.023), and magnesium (*p* = 0.046) after adjusting the model for age, sex, and BMI.

## 4. Discussion

We present, to our knowledge, the first study that evaluates adherence to the GFD in newly diagnosed patients with CD (<6 months). Our results suggest that early follow-up of the newly diagnosed CD is essential, both to assess adherence to the GFD, which is the only treatment currently available for the disease and to determine the GFD nutritional adequacy. It is unclear which is the most effective method to assess adherence to the GFD, and in this regard, our study demonstrates the role of stool GIP determination in the early monitoring of the GFD. On the other hand, it emphasizes the importance of nutritional intervention in the follow-up of these patients, as it has been shown that many patients with CD on GFD have nutritional imbalances and macronutrient and micronutrient deficits.

Adherence to the GFD is essential for mucosal recovery in newly diagnosed patients with CD. Non-adherence to the gluten-free diet can be due to different reasons. One of them may be due to the change in eating behaviour that occurs in the patient who changes his habits towards a GFD. Previous studies [36,37] have shown that a GFD leads to a higher intake of fat, protein, and UPF, although it has not yet been established whether nutritional imbalances have an impact on adherence to the GFD. In our cohort, there was no difference between patients with GIP+ and those with GIP−, suggesting that adherence is not influenced by dietary fat and protein intake, which is a common problem at the start of the diet. Determination of GIP in stool is a non-invasive method that provides direct information on recent gluten exposure [9]. In our study, we have assessed adherence to the GFD by determining GIP excreted in stool in a cohort of children with newly diagnosed CD. To identify intermittent gluten intakes, we carried out double determinations 4 months after CD diagnosis, indirectly analyzing the weekend diet and the weekday’s diet, thus allowing the situation to be like what happens in real life. Ruiz-Carnicer et al. [3] and Stefanolo et al. [20] have already described the importance of multiple determinations to increase the sensitivity and specificity of the detection of GIPs in urine and stool, respectively. Our study reveals poor dietary compliance rates of 14% at 4 months, based on stool GIPs. Gerasimidis et al. [26] found 16% positive stool GIP in pediatric celiac patients who reported good GFD compliance after 6 months of GFD, while Comino et al. [19] demonstrated a stool GIP positivity of 23% in celiac children after 6 months of GFD. Fernandez et al. [38] found 92.5% GFD adherence as determined by stool GIPs, with a large percentage of patients with positive stool GIPs but negative serological controls (anti-transglutaminase antibodies); in this study, lower GFD adherence was reported with increasing patient age and time since diagnosis of CD. Other authors have also described a worsening of adherence with longer disease progression [39]. In the long term, several aspects have been identified as the factors influencing this poor adherence to the GFD, such as less parental supervision (greater autonomy of patients, especially adolescents, who also eat more likely outside the home [19,40]), less awareness of the disease as symptomatology improves with adequate initial GFD adherence, and in some cases, the psychological overload of following a strict diet [19], especially in adolescents and young adults. In newly diagnosed celiac patients, especially in the pediatric population, these aspects should not be present, thus there is the need to emphasize appropriate nutritional counselling for patients and their families at the time of diagnosis, clear any doubts that arise in the process, and ensure early follow-up of celiac children after CD diagnosis. Interestingly, our study has identified the age group with the highest percentage of poor adherence to the GFD based on the determination of stool GIPs (under 2 years of age), as opposed to previous studies [19], and this may contribute to the development of age-specific follow-up strategies.

The assessment of positive GIPs together with a dietary record concomitant with stool sample collection allows the development of dietary interventions aimed at improving GFD adherence. In this regard, close counselling by a dietitian can improve GFD adherence, as demonstrated by the determination of GIP in control stool at 9 months in patients who were initially GIP+. Our results showed that 88% of those did not perform properly. GFDs were compliers after a nutritional education session. Regarding the consumption of UPF, we did not found differences between patients with GIP+ vs. GIP−, so we can speculate that the consumption of UPF does not allow to distinguish patients who adequately follow the diet. In this regard, it is important to emphasize the importance of appropriate initial advice to patients and families, providing them with information on the variety of gluten-free products available, especially natural gluten-free foods.

A GFD should be a balanced diet, which allows for optimal growth and development of children with CD. Nutritional imbalances have been described in patients with CD on GFD, including micronutrients (magnesium, calcium, iron, zinc, B vitamins, vitamin D, and folic acid) and fiber deficiencies. In our study, we observed deficient intake of vitamin D (only 27% of patients have an adequate intake, a similar percentage to that described in other studies in celiac patients, both in the diet prior to CD diagnosis and in the GFD [22]), as well as folate, calcium, and magnesium. We have also verified an inadequate fiber intake (73% adequacy), in line with other studies in patients with CD [22,24,41]. These findings suggest that nutritional counselling should be a priority for these patients at the beginning of the GDF, and early follow-up is essential to detect deficiencies that need to be corrected. In addition, we have also reported macronutrient imbalances in patients on a GFD. In our study, 66% of patients exceeded the recommended fat intake, and all of them exceeded the recommended protein intake [35]. Furthermore, a high percentage of total daily energy intake came from UPF, with these foods being mostly consumed in the morning and afternoon snacks. Other studies [42] have already demonstrated this high consumption of UPF, which may be due to several factors: on the one hand, the lower cost of manufactured gluten-free products compared to naturally gluten-free foods; on the other hand, the greater palatability and aesthetic appeal of the manufactured gluten-free products, especially for children; furthermore, families’ lack of awareness of the different options available at the start of the GFD [42,43]. In our cohort, patients who consumed more energy from UPF had poor diet quality, as previously described by our research group [42].

Excess protein and fat intake in the diet of these patients, as well as high UPF consumption, has been linked to the development of other long-term health problems. On the one hand, excess fat and protein intake may contribute to the development of overweight or obesity, resulting in additional problems. Kabbani et al. [44] described an increase in the BMI in celiac patients over the course of the disease, especially in patients with higher GFD adherence: 4.4% of patients with low weight at diagnosis developed overweight or obesity, 17% of patients with normal weight at diagnosis developed overweight or obesity, and 17.3% of patients with overweight at diagnosis became obese. In addition, higher UPF consumption has been associated with an increased risk of overweight, obesity, metabolic syndrome, dyslipidemia, functional gastrointestinal disorders (irritable bowel syndrome and functional dyspepsia), recurrent wheezing, and depression in adult patients [45] and alterations of the gut microbiota [46]. All these aspects mostly lead to a worsening of digestive symptoms as well as the quality of life of these patients. This is another reason why action protocols should focus, among others, on nutritional education, to improve the quality of life and health of patients in the long term.

## 5. Strengths and Limitations

There are several limitations that should be highlighted. Firstly, this is a cross-sectional pilot study with a relatively small sample size, so results must be interpreted with caution. However, we present the first study to assess adherence to the GFD at such an early stage. Secondly, another limitation of our study is the lack of a control group, which is difficult given the assessment of a specific type of diet in the group of patients with CD. Thirdly, there has been no follow-up of the cohort, so we have not been able to evaluate the diet quality in the medium and long term; however, the aim of our study was to assess early disbalances resulting from the implementation of the GFD. It would be advisable to take these limitations into account and to recruit a larger number of patients.

## 6. Conclusions

The findings of our study demonstrate the role of stool GIP determination in monitoring GFD adherence in the first months after CD diagnosis, which may be key in the early detection of patients with transgressions or inadvertent gluten intakes. This may allow the development of specific follow-up strategies based on the initial determination of GIPs, as well as clinical management protocols. More studies are needed to correlate GIP positivity with serology, as well as studies with a control group, to increase the validity of the results. On the other hand, early monitoring of the diet of patients with CD was able to detect important nutritional imbalances and deficiencies, thus guiding the dietary advice of our celiac patients more precisely to prevent future nutritional diseases. We believe that it is important to highlight the participation of dietitians in the management of the disease to guide the GFD.

## Figures and Tables

**Table 1:** Characteristics of the study participants (n = 61).

Variable	Mean (SD)
Age (years)	7.5 (3.9)
Sex (n [%])	
Male	24 (39.3)
Female	37 (60.7)
Weight (kg)	25.9 (13.3)
Height (m)	1.2 (0.2)
Body mass index categories (n [%])	
Underweight	2 (3.3)
Normoweight	44 (72.1)
Overweight	11 (18.0)
Obese	4 (6.6)
Energy (kcal) (n = 58)	1543.9 (462.2)
Fat (% of total energy intake)	38.2 (6.7)
Protein (% of total energy intake)	16.0 (4.0)
Carbohydrates (% of total energy intake)	44.2 (6.6)
NOVA food classification (n = 58)	
Unprocessed or minimally processed foods (kcal/day)	601.0 (215.8)
Unprocessed or minimally processed foods (%E)	39.8 (11.5)
Processed culinary ingredients (kcal/day)	102.3 (63.4)
Processed culinary ingredients (%E)	6.7 (3.4)
Processed Foods (kcal/day)	98.1 (84.1)
Processed Foods (%E)	6.2 (4.9)
Ultra-processed food and drink products (kcal)	739.4 (316.7)
Ultra-processed food and drink products (%Energy)	47.5 (13.1)
Fecal gluten immunogenic peptides (n [%]) (n = 60)	
Positive	8 (13.3)
Negative	52 (86.7)
Other diseases (yes, n [%])	4 (6.6)

Values shown as mean (standard deviation) unless otherwise indicated.

**Table 2:** Differences in dietary intake by fecal gluten immunogenic peptide detection (negative vs. positive).

	Negative (n = 49)	Positive (n = 8)	*p* ^a^	*p* ^b^
Dietary intake				
Energy (kcal)	1559.3 (65.1)	1568.4 (161.1)	0.958	0.446
Fat (% of E)	38.9 (0.7)	38.7 (1.8)	0.929	0.953
Protein (% of E)	16.1 (0.6)	15.3 (1.4)	0.620	0.690
Carbohydrates (% E)	43.9 (0.9)	45.1 (2.4)	0.669	0.649
Fiber (g/day)	10.5 (0.7)	9.6 (1.7)	0.609	0.933
Sugar (g/day)	22.4 (2.0)	16.8 (5.2)	0.342	0.525
Vitamin A (µg)	320.2 (19.0)	274.6 (47.1)	0.373	0.493
Vitamin D (µg)	3.8 (0.7)	2.2 (1.8)	0.435	0.564
Vitamin E	4.4 (0.3)	5.2 (0.7)	0.380	0.234
Riboflavin (mg)	0.9 (0.05)	1.1 (0.1)	0.291	0.395
Folate (µg)	97.4 (6.3)	99.3 (15.5)	0.911	0.747
Cobalamin (µg)	4.2 (0.5)	3.3 (1.1)	0.511	0.609
Calcium (mg)	500.4 (38.5)	605.2 (95.4)	0.313	0.339
Vitamin C (mg)	46.1 (6.2)	31.8 (15.3)	0.390	0.721
Potasium (mg)	1496.8 (88.8)	1569.2 (219.7)	0.761	0.582
Magnesium (mg)	119.4 (6.7)	129.5 (16.5)	0.572	0.421
NOVA food classification				
Unprocessed or minimally processed foods (%E)	39.3 (1.7)	42.5 (4.1)	0.472	0.447
Processed culinary ingredients (%E)	6.7 (0.5)	7.6 (1.2)	0.489	0.463
Processed Foods (%E)	5.8 (0.7)	7.6 (1.7)	0.335	0.285
Ultra-processed food and drink products (%Energy)	48.6 (1.9)	41.3 (4.6)	0.146	0.088

^a^ Model unadjusted ^b^ Model adjusted for age, sex, and body mass index. D, day; S, servings; W, week.

## Data Availability

The data presented in this study are available on request from the corresponding author.

## Supplementary Materials

The following are available online at https://www.mdpi.com/article/10.3390/nu15071761/s1, Supplementary Figure S1: Percentage of adequacy for energy intake, fiber, protein, and micronutrients recommended daily intake in children with celiac disease according to fecal detectable gluten immunogenic peptides (n = 58). Values are shown as mean (standard error). Model is adjusted for age, sex, and body mass index.
