# Supplementary material for: The Importance of an Early Evaluation after Establishing a Gluten-Free Diet in Children with Celiac Disease
Source: Nutrients. 2023 Apr 4;15(7):1761. doi: 10.3390/nu15071761 (PMC10097184; doi:10.3390/nu15071761)
Supplement: Supplementary file 1 [file nutrients-15-01761-s001.zip › nutrients-2280097-supplementary.pdf]

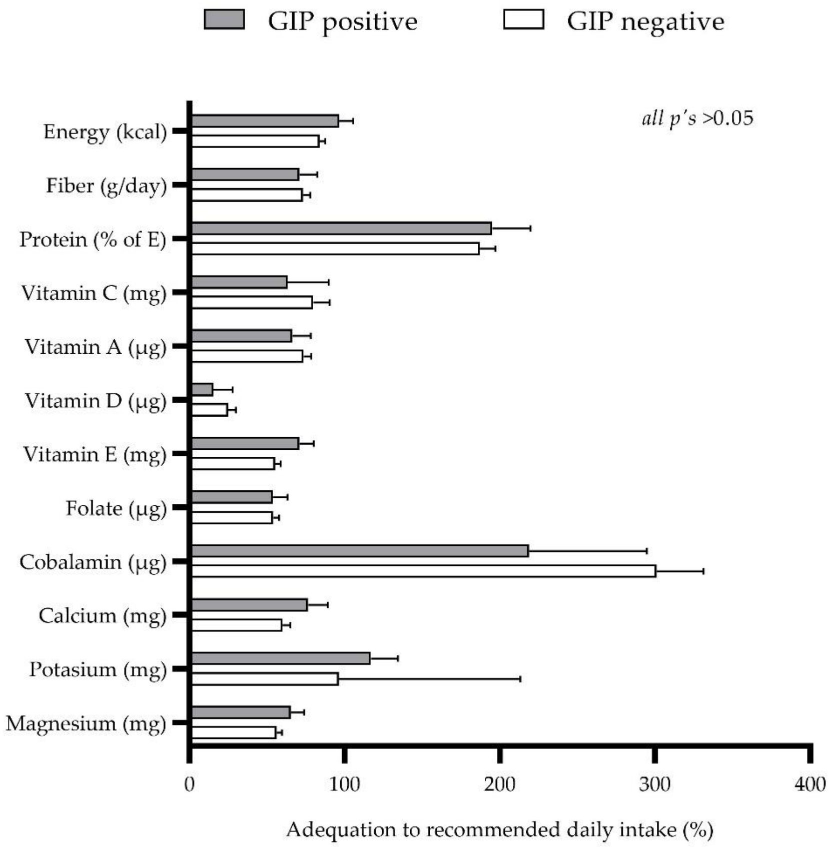

Supplementary Figure S1. Percentage of adequacy for energy intake, fiber, protein and micronutrients recommended daily intake in children with celiac disease according to fecal detectable gluten immunogenic peptides (n=58). Values shown as mean (standard error). Model adjusted for age, sex and body mass index.
